# Induction of apoptosis in HCT-116 colon cancer cells by polysaccharide of *Larimichthys crocea* swim bladder

**DOI:** 10.3892/ol.2014.2756

**Published:** 2014-12-02

**Authors:** HUAYI SUO, JIA-LE SONG, YALIN ZHOU, ZHENHU LIU, RUOKUN YI, KAI ZHU, JIE XIE, XIN ZHAO

**Affiliations:** 1 College of Food Science, Southwest University, Chongqing 400715, P.R. China; 2 Department of Nutrition and Food Hygiene, School of Public Health, Guilin Medical University, Guilin, Guangxi 541004, P.R. China; 3 Department of Food Science and Nutrition, Pusan National University, Busan 609735, Republic of Korea; 4 Department of Biological and Chemical Engineering, Chongqing University of Education, Chongqing 400067, P.R. China; 5 Science and Technology Administration, Chinese Academy of Agricultural Sciences, Beijing 100081, P.R. China; 6 Institute of Functional Ecological Food, Chongqing University of Education, Chongqing 400067, P.R. China

**Keywords:** polysaccharide, *Larimichthys crocea* swim bladder, HCT-116 human colon cancer cells, apoptosis, gene

## Abstract

*Larimichthys crocea* swim bladder is a traditional food and medicine widely used in China. The *in vitro* anticancer effects of polysaccharide of *L. crocea* swim bladder (PLCSB) in HCT-116 human colon cancer cells was investigated by 3-(4,5-dimethylthiazol-2-yl)-2,5-diphenyltetrazolium bromide assay. At concentrations ranging between 0 and 800 μg/ml PLCSB, cancer cell viability was decreased by PLCSB in a concentration-dependent manner. In particular, 400 μg/ml PLCSB significantly (P<0.05) induced apoptosis, which was demonstrated by 4,6-diamidino-2-phenylindole staining and flow cytometry analysis. To elucidate the mechanisms underlying the anticancer effect of PLCSB in HCT-116 cancer cells, the expression of apoptosis and metastasis-associated genes was analyzed by reverse transcription-polymerase chain reaction and western blot analysis. A total of 400 μg/ml PLCSB significantly induced apoptosis in HCT-116 cells (P<0.05) via the upregulation Bax, p53, p21, apoptotic protease activating factor 1, caspase-3, -8, and -9, as well as Fas and the downregulation of B-cell lymphoma 2 (Bcl-2), Bcl-extra large and Fas ligand (L). The results of this study demonstrated that PLCSB exhibits an anticancer effect on HCT-116 colon cancer cells, *in vitro*.

## Introduction

The swim bladder is important organ in Osteichthyes that is used to maintain balance and contains ~10% polysaccharide. *Larimichthys crocea* is used as drug in traditional Chinese medicine (TCM) and it has been reported that *L. crocea* swim bladder may remove free radicals and protect against cancer (1). Polysaccharides are an important material for producing drugs, and the polysaccharide obtained from *Phellinus linteus* and *Pleurotus ostreatus* have been shown to exhibit an anticancer effect in colon cancer cells *in vitro* (2,3).

Apoptosis induction in cancer cells is characterized by changes in cell morphology, which include cell shrinkage, membrane blebbing, chromatin condensation and nuclear fragmentation (4). Apoptosis presents a critical defense mechanism against cancer, which leads to the death of potentially harmful cells. Dysregulated apoptotic processes have been implicated in numerous diseases; these lead to the inhibition of cell death and the progression of diseases, including cancer (5). Identifying the critical events involved with carcinogenesis may present an opportunity to prevent cancer development using TCM that triggers apoptosis, particulary with the extraction of natural substances. However, TCM may augment disease progression. In addition to the effects on protein expression and function, an increasing number of studies have reported that a large number of components of TCM may exert effects on the human genome, via the direct or indirect modulation of gene expression (6).

In the present study, the *in vitro* anticancer effects of polysaccharide of *L. crocea* swim bladder (PLCSB) were determined by 3-(4,5-dimethylthiazol-2-yl)-2,5-diphenyltetrazolium bromide (MTT) assay, 4,6-diamidino-2-phenylindole (DAPI) staining test, flow cytometry analysis, mRNA and protein expression analysis, and the molecular mechanisms underlying the anticancer effects of PLCSB were also investigated.

## Materials and methods

### PLCSB preparation

Wild Yellow Sea *L. crocea* were purchased from Shandong Linyi Dahai Aquaculture Company (Linyi, China). The swim bladder of *L. crocea* (1 kg) was freeze-dried and the samples were crushed. A total of 3 l petroleum ether was mixed with the swim bladder of *L. crocea* and reflux extraction was performed twice, for 1 h at 60°C to remove the protein. Next, the residue was collected following filtration. A total of 3 l absolute ethyl alcohol was then added and reflux extraction was performed for 3 h, and the residue without protein was filtrated and collected. Finally, 3 l water was added and the residue was extracted at 60°C for 2 h and the filter liquid was collected (7). The crude PLCSB was obtained following evaporation.

### Cancer cell preparation

HCT-116 human colon carcinoma cells and human HaCaT keratinocyte cells were purchased from the American Type Culture Collection (Manassas, VA, USA). The HCT-116 cells were cultured in RPMI-1640 medium (Gibco-BRL, Carlsbad, CA, USA) and HaCaT cells were cultured in Dulbecco’s modified Eagle’s medium (Gibco-BRL) supplemented with 10% fetal bovine serum (Gibco-BRL) and 1% penicillin-streptomycin (Gibco-BRL) at 37°C in a humidified atmosphere containing 5% CO_2_ (Forma 311 S/N29035 CO_2_ incubator; Forma Therapeutics, Inc., Watertown, MA, USA). The medium was changed two-three times a week.

### MTT assay

The anticancer effects of PLCSB were assessed by MTT assay. HCT-116 cells were seeded in a 96-well plate (2×10^4^ cells/ml per well) in a volume of 180 μl. Next, 20 μl of 100, 200 or 400 μg/ml PLCSB were added. The cells were then incubated with the PLCSB solutions for 48 h at 37°C in an incubator (311 S/N29035; Forma Therapeutics, Inc.) in a humidified atmosphere containing 5% CO_2_. An MTT solution (200 μl; 5 mg/ml; Amresco LLC, Solon, OH, USA) was added to each well and the cells were cultured for an additional 4 h under the same conditions. The supernatant was discarded and 150 μl dimethyl sulfoxide (per well) was added and mixed for 30 min. Finally, the absorbance of each well was measured by an ELISA plate reader (Bio-Rad 680; Bio-Rad, Hercules, CA, USA) at a wavelength of 540 nm (8).

### DAPI staining

Untreated control cells and cells treated with different concentrations of PLCSB were harvested, washed with phosphate-buffered saline (PBS), and fixed with 3.7% paraformaldehyde (Sigma-Aldrich, St. Louis, MO, USA) in PBS for 10 min at room temperature. The fixed cells were then washed with PBS and stained with a 1 mg/ml DAPI (Sigma-Aldrich) solution for 10 min at room temperature (9). The cells were washed twice with PBS and examined with a fluorescence microscope (BX50; Olympus Corporation, Tokyo, Japan).

### Flow cytometry analysis

For histological analysis, liver tissues were fixed in 10% (v/v) buffered formalin for 24 h, dehydrated in ethanol and embedded in paraffin. Next, 4-μm-thick sections were prepared and stained with hematoxylin and eosin, and then observed under a microscope (BX41; Olympus Corporation) (10).

### Reverse transcription-polymerase chain reaction (RT-PCR) analysis

Total RNA was extracted from cancer cells using TRIzol reagent (Invitrogen Life Technologies, Carlsbad, CA, USA) according to the manufacturer’s instructions. RNA was digested by RNase-free DNase (Roche Diagnostics, Basel, Switzerland) for 15 min at 37°C and purified using an RNeasy kit (Qiagen, Hilden, Germany) according to the manufacturer’s instructions. cDNA was synthesized from 2 μg of total RNA by incubation at 37°C for l h with avian myeloblastosis virus reverse transcriptase (GE Healthcare Life Sciences, Chalfont, UK) according to the manufacturer’s instructions. The primer sequences used to amplify the genes were as follows: Forward, 5′-AAGCTGAGCGAGTGTCTCCGGCG-3′ and reverse, 5′-CAGATGCCGGTTCAGGTACTCAGTC-3′ for Bax; forward, 5′-CTCGTCGCTACCGTCGTGACTTGG-3′ and reverse, 5′-CAGATGCCGGTTCAGGTACTCAGTC-3′ for B-cell lymphoma 2 (Bcl-2); forward, 5′-CAGCTGCACCTGACG-3′ and reverse, 5′-GCTGGGTAGGTGCAT-3′ for Bcl-extra large (xL); forward, 5′-GCTCTGACTGTACCACCATCC-3′ and reverse, 5′-CTCTCGGAACATCTCGAAGCG-3′ for p53; forward, 5′-CTCAGAGGAGGCGCCATG-3′ and reverse, 5′-GGGCGGATTAGGGCTTCC-3′ for p21; forward, 5′-CTATGAGCTAGTCATGTGTTAGA-3′ and reverse, 5′-CCAATTCACAGACACTGACA-3′ for apoptotic protease activating factor 1 (Apaf-1); forward, 5′-CAAACTTTTTCAGAGGGGATCG-3′ and reverse, 5′-GCATACTGTTTCAGCATGGCA-3′ for caspase-3; forward, 5′-CTGCTGGGGATGGCCACTGTG-3′ and reverse, 5′-TCGCCTCGAGGACATCGCTCTC-3′ for caspase-8; forward, 5′-GGCCCTTCCTCGCTTCATCTC-3′ and reverse, 5′-GGTCCTTGGGCCTTCCTGGTAT-3′ for caspase-9; forward, 5′-GAAATGAAATCCAAAGCT-3′ and reverse, 5′-TAATTTAGAGGCAAAGTGGC-3′ for Fas; and forward, 5′-GGATTGGGCCTGGGGATGTTTCA-3′ and reverse, 5′-TTGTGGCTCAGGGGCAGGTTGTTG-3′ for Fas ligand (L). Glyceraldehyde 3-phosphate dehydrogenase was amplified as an internal control gene using the following primers: Forward, 5′-CGGAGTCAACGGATTTGGTC-3′ and reverse, 5′-AGCCTTCTCCATGGTCGTGA-3′. Amplification was performed in a thermal cycler (Mastercycler Nexus X1; Eppendorf, Hamburg, Germany). The PCR products were separated in 1.0% agarose gels and visualized with ethidium bromide staining (11).

### Western blot analysis

Total cell lysates were obtained using extraction buffer as previously described (12). Protein was extracted using cell lysis solution (WB-0061, Beijing Dingguo Changsheng Biotechnology Co. Ltd., Beijing, China) and protein concentrations were determined using a protein assay kit (Bio-Rad). For western blot analysis, aliquots of lysate containing 30–50 μg of protein were separated by electrophoresis on 12% sodium dodecyl sulfate-polyacrylamide gels and then electrotransferred onto a nitrocellulose membrane (Schleicher & Schuell BioScience, Inc., Keene, NH, USA). After blocking with skimmed milk, the membranes were probed with specific primary antibodies for 1 h and then incubated with the appropriate horseradish peroxidase-conjugated polyclonal secondary antibodies (goat anti-human; ab6958; dilution, 1:1,000; Abcam, Cambridge, UK). Primary antibodies included mouse anti-human Bax monoclonal antibody (sc-65532; dilution, 1:200), mouse anti-human Bcl-2 monoclonal antibody (sc-509; dilution, 1:200), mouse anti-human Bcl-xL monoclonal antibody (sc-136207; dilution, 1:100), mouse anti-human p53 monoclonal antibody (sc-55476; dilution, 1:200), mouse anti-human p21 monoclonal antibody (sc-56335; dilution, 1:200), goat anti-human Apaf-1 polyclonal antibody (sc-33870; dilution, 1:200), mouse anti-human caspase-3 polyclonal antibody (sc-56052; dilution, 1:200), mouse anti-human caspase-8 monoclonal antibody (sc-81657; dilution, 1:200), mouse anti-human caspase-9 monoclonal antibody (sc-56073; dilution, 1:200) (Santa Cruz Biotechnology, Inc., Santa Cruz, CA, USA), rabbit anti-human Fas polyclonal antibody (ab82419; dilution, 1:1,000), rabbit anti-human FasL polyclonal antibody (ab15285; dilution, 1:200) and rabbit anti-human β-actin polyclonal antibody (ab16039; dilution, 1:200) (Abcam). Antibody binding was visualized by enhanced chemiluminescence according to the manufacturer’s instructions (GE Healthcare). Chemiluminescence was visualized using a LAS3000 luminescent image analyzer (Fujifilm Corporation, Tokyo, Japan).

### Statistical analysis

Data are presented as the mean ± standard deviation. Differences between individual groups were assessed by one-way analysis of variance and Duncan’s multiple range test. P<0.05 was considered to indicate a statistically significant difference. SAS version 9.1 software (SAS Institute Inc., Cary, NC, USA) was used for statistical analyses.

## Results

### Inhibitory effects of PLCSB on cancer cell growth

**Figure 1:**
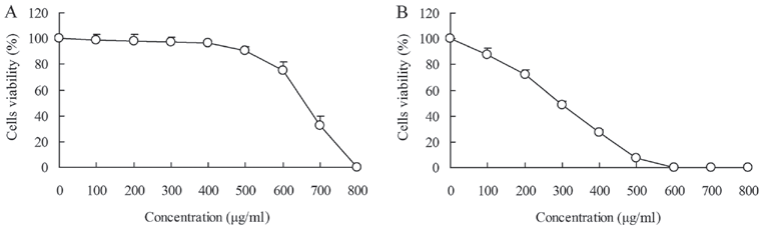
Effect of polysaccharide of *Larimichthys crocea* swim bladder on the growth of (A) human HaCaT keratinocyte cells and (B) HCT-116 human colon cancer cells.

The inhibitory effects of PLCSB on HCT-116 cell growth were analyzed by MTT assay. HaCaT keratinocyte cells were evaluated and the growth inhibitory rates were not associated with the concentration of PLCSB. When the cells were treated with 0–400 μg/ml PLCSB, no significant difference in the growth inhibitory rates were identified, and the rates were all <10%. When the cells were treated with >400 μg/ml PLCSB, the growth inhibitory rate increased and, following treatment with 800 μg/ml PLCSB, the rate was 100% (Fig. 1A). At concentrations ranging between 0 and 400 μg/ml, cell viability was decreased by the PLCSB in a concentration-dependent manner. At the concentration of 600 μg/ml, the survival rate of the cells treated with PLCSB reached 0% (Fig. 1B). No inhibitory or toxic effects on the growth of normal human cells were observed at concentrations of 0–400 μg/ml PLCSB; however, an inhibitory effect on growth was exhibited in HCT-116 cancer cells. These results indicated that PLCSB only exerts an effect on cancer cells. Consequently, concentrations of 100, 200 and 400 μg/ml PLCSB were selected for subsequent experiments.

### Induction of apoptosis by PLCSB

**Figure 2:**
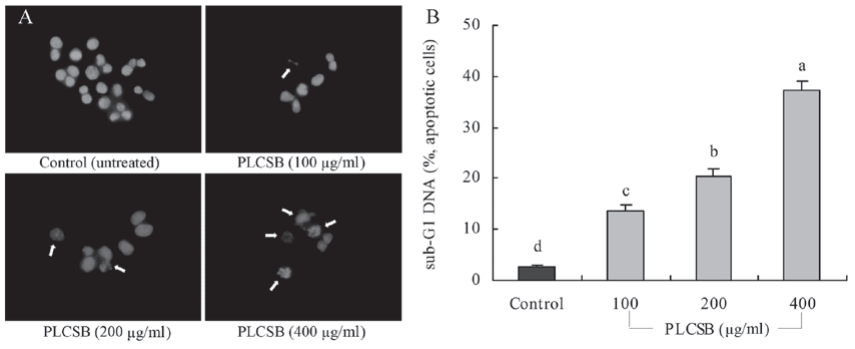
Exposure of HCT-116 human colon carcinoma cells to PLCSB induces apoptosis. (A) The appearance of apoptotic bodies in HCT-116 cells treated with PLCSB was determined by 4,6-diamidino-2-phenylindole assay. (B) Treatment with HCT-116 increased the number of apoptotic cells as measured by flow cytometry. The profile represents an increased sub-G1 population (apoptotic cells). PLCSB, polysaccharide of *Larimichthys crocea* swim bladder.

To determine a possible mechanism underlying the growth inhibitory activity of PLCSB in HCT-116 cancer cells, the induction of apoptosis was analyzed. The extent of chromatin condensation was determined by fluorescence microscopy of the cells stained with the DNA-binding fluorescent dye DAPI and flow cytometric analysis. While the untreated HCT-116 cells exhibited nuclei with homogeneous chromatin distribution, treatment with the PLCSB induced chromatin condensation and nuclear fragmentation, which indicated the presence of apoptotic cells (Fig. 2A). Chromatin condensation and the formation of apoptotic bodies, which are two hallmarks of apoptosis, were observed in cells cultured with 200 and 400 μg/ml PLCSB. By contrast, the level of chromatin condensation was low in the cells treated with 100 μg/ml PLCSB. Flow cytometric analyses revealed that treatment with PLCSB promoted apoptosis of the HCT-116 cells, compared with the untreated control cancer cells. This conclusion is based on the significant accumulation of cells with a sub-G1 DNA content (Fig. 2B). The induction of apoptosis was almost negligible (2.7%) in untreated control cancer cells; however, cancer cells treated with 400 μg/ml PLCSB exhibited a higher level of apoptosis (37.2%) than cells treated with 100 μg/ml (13.7%) and 200 μg/ml (20.5%) PLCSB.

### Bcl-2 family gene expression

**Figure 3:**
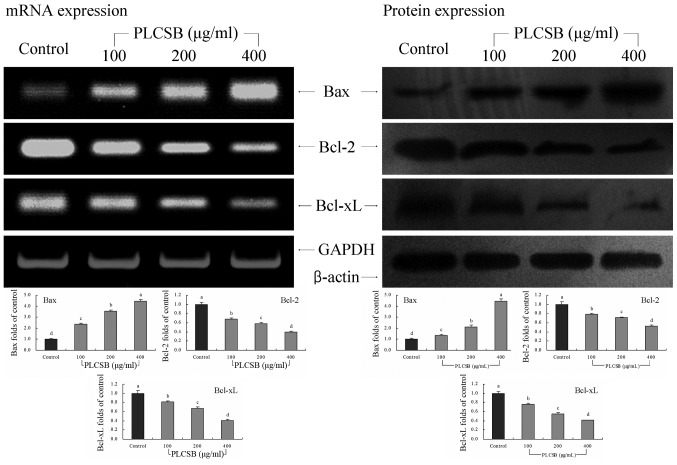
Effects of PLCSB on the mRNA and protein expression of Bax, Bcl-2 and Bcl-xL in HCT-116 human colon cancer cells. Band intensity was measured with a densitometer and expressed as relative to the untreated control. Fold ratio = gene expression/GAPDH (β-actin) × control numerical value (control fold ratio: 1). ^a–d^ Mean values with different letters over the bars are significantly different (P<0.05) according to Duncan’s multiple-range test. PLCSB, polysaccharide of *Larimichthys crocea* swim bladder; Bcl-2, B-cell lymphoma 2; xL, extra large; GAPDH, glyceraldehyde 3-phosphate dehydrogenase.

To investigate the mechanisms underlying the inhibition of cancer cell growth by PLCSB, the expression of Bax, Bcl-2 and Bcl-xL in HCT-116 human colon cancer cells was analyzed by RT-PCR and western blot analysis following incubation with 100, 200 or 400 μg/ml PLCSB for 48 h. The expression of pro-apoptotic Bax and anti-apoptotic Bcl-2 and Bcl-xL exhibited significant changes (P<0.05) following treatment with PLCSB (Fig. 3). These results indicate that treatment with PLCSB leads to apoptosis induction in HCT-116 cells via a Bax-, Bcl-2- and Bcl-xL-dependent pathway.

### p53 and p21 gene expression

**Figure 4:**
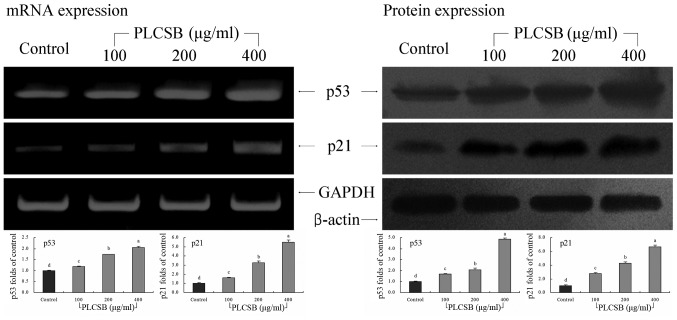
Effects of PLCSB on the mRNA and protein expression of p53 and p21 in HCT-116 human colon cancer cells. Band intensity was measured with a densitometer and expressed as relative to the untreated control. Fold ratio = gene expression/GAPDH (β-actin) × control numerical value (control fold ratio: 1). ^a–d^Mean values with different letters over the bars are significantly different (P<0.05) according to Duncan’s multiple-range test. PLCSB, polysaccharide of *Larimichthys crocea* swim bladder; GAPDH, glyceraldehyde 3-phosphate dehydrogenase.

As shown in Fig. 4, PLCSB significantly increased the level of p53 and p21 mRNA expression (P<0.05). These changes in p53 and p21 expression as a result of PLCSB treatment may lead to the induction of apoptosis in HCT-116 cells. These results showed that PLCSB exhibits significant anticancer activity via the induction of apoptosis.

### Apaf-1 gene expression

**Figure 5:**
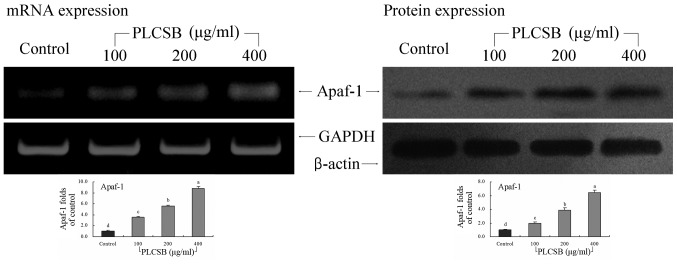
Effects of PLCSB on the mRNA and protein expression of Apaf-1 in HCT-116 human colon cancer cells. Band intensity was measured with a densitometer and expressed as relative to the untreated control. Fold ratio = gene expression/GAPDH (β-actin) × control numerical value (control fold ratio: 1). ^a–d^Mean values with different letters over the bars are significantly different (P<0.05) according to Duncan’s multiple-range test. PLCSB, polysaccharide of *Larimichthys crocea* swim bladder; GAPDH, glyceraldehyde 3-phosphate dehydrogenase; Apaf-1, apoptotic protease activating factor 1.

The mRNA and protein expression of Apaf-1 was increased by treatment with PLCSB, with the greatest Apaf-1 expression observed in the 400-μg/ml PLCSB-treated cells (8.85- and 6.45-fold that of the mRNA and protein expression in the untreated cancer cells, respectively) (Fig. 5). The Apaf-1 mRNA and protein expression in 200-μg/ml PLCSB-treated cells was 5.57- and 3.90-fold that of the control cells. The 100-μg/ml PLCSB-treated cells also showed higher Apaf-1 mRNA and protein expression than the control cells (3.59- and 1.94-fold, respectively), but this was lower than that of cells treated with 200 and 400 μg/ml PLCSB.

### Caspase gene expression

**Figure 6:**
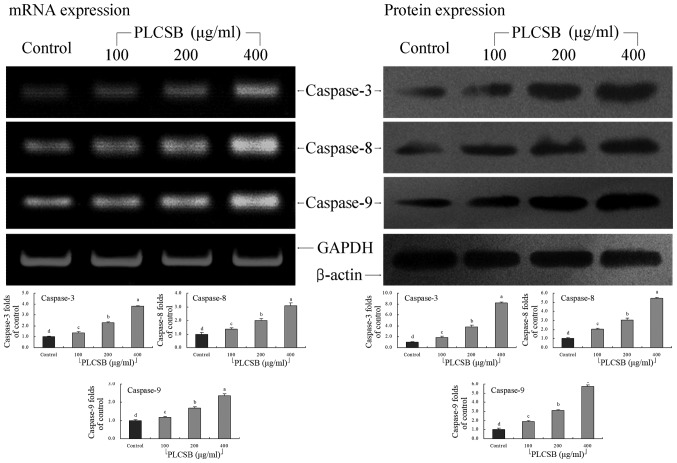
Effects of PLCSB on the mRNA and protein expression of caspase-3, -8 and -9 in HCT-116 human colon cancer cells. Band intensity was measured with a densitometer and expressed as relative to the untreated control. Fold ratio = gene expression/GAPDH (β-actin) × control numerical value (control fold ratio: 1). ^a–d^Mean values with different letters over the bars are significantly different (P<0.05) according to Duncan’s multiple-range test. PLCSB, polysaccharide of *Larimichthys crocea* swim bladder; GAPDH, glyceraldehyde 3-phosphate dehydrogenase.

The mRNA expression levels of caspase-3, -8 and -9 were extremely low in untreated control HCT-116 cells; however, the levels significantly increased following treatment with 400 μg/ml PLCSB (P<0.05). Following PLCSB treatment, the mRNA expression of caspase-3, -8 and -9 were gradually increased in a dose-dependent manner (Fig. 6). Furthermore, the induction of apoptosis by PLCSB was associated with the upregulation of caspase-3, -8 and -9 mRNA and protein expression.

### Fas and FasL gene expression

**Figure 7:**
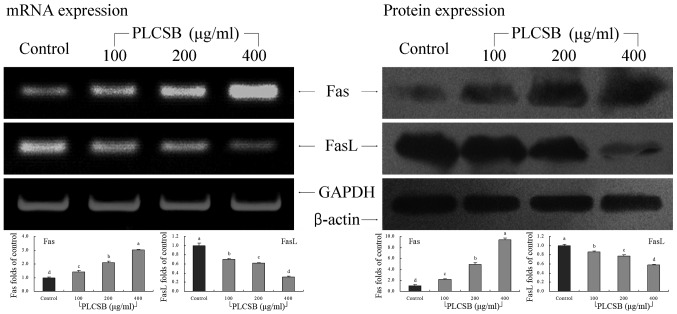
Effects of PLCSB on the mRNA and protein expression of Fas and FasL in HCT-116 human colon cancer cells. Band intensity was measured with a densitometer and expressed as relative to the untreated control. Fold ratio = gene expression/GAPDH (β-actin) × control numerical value (control fold ratio: 1). ^a–d^Mean values with different letters over the bars are significantly different (P<0.05) according to Duncan’s multiple-range test. PLCSB, polysaccharide of *Larimichthys crocea* swim bladder; GAPDH, glyceraldehyde 3-phosphate dehydrogenase; FasL, Fas ligand.

This study further determined whether the apoptosis-inducing actions of PLCSB were associated with inhibition of Fas and FasL gene expression. As shown in Fig. 7, PLCSB demonstrated induction activity of apoptosis in HCT-116 cells, as indicated by increased mRNA and protein expression of Fas along with decreased FasL expression when compared with untreated cancer cells (P<0.05).

## Discussion

The swim bladder has been historically used as a folk medicine. Recently, swim bladder has been shown to alleviate various inflammatory conditions, and it may also augment the function of platelets, capillary vessels and clotting factors (11). Polysaccharides are the main component of swim bladder; however, few studies have investigated the polysaccharides of the swim bladder, and these studies showed that polysaccharides of the swim bladder posses anti-inflammatory effects (12,13). To the best of our knowledge, the present study was the first to investigate the anticancer effect of apoptosis induction by PLCSB *in vitro.* The results demonstrated that the PLCSB exhibited a marked apoptosis-inducing effect against the HCT-116 colon cancer cells.

Apoptosis induction in cancer cells is a potentially promising approach for cancer therapy (14). In the present study, PLCSB decreased the growth of HCT-116 cells via the induction of apoptosis. Apoptotic cells with degraded DNA exhibit hypodiploid DNA content and are presented as sub-G1 peaks on DNA histograms, which are used to count the percentage of apoptotic cells (15). The formation of apoptotic bodies was observed, in addition to increased sub-G1 DNA (apoptotic cells) accumulation in cells treated with PLCSB.

Apoptosis is a critical cellular event and, thus, eludicating its mechanisms of action may present potential for improvements in tumor diagnosis and therapy (16). In normal cells, the anti-apoptotic protein Bcl-2 is expressed on the outer mitochondrial membrane surface (17). The apoptosis regulator BAX promotes apoptosis by binding to and antagonizing the Bcl-2 protein (18). Bcl-xL is a member of the Bcl-2 protein family and it is hypothesized that the relative amount of pro- and anti-survival Bcl-2 family of proteins determines whether a cell undergoes apoptosis (19). The Bax, Bcl-2 and Bcl-xL genes are predominantly expressed during apoptosis and, thus, the effects of these genes on apoptotic activity were determined.

p53, which is a tumor supressor, upregulates expression of the Bax protein, which has been found to be involved in p53-mediated apoptosis (20). p53 is a transcription factor, which regulates the Bax downstream target gene when activated in response to stress (21). The expression of the p21 gene is tightly controlled by the tumor suppressor protein p53, through which this protein mediates the p53-dependent cell cycle G1 phase arrest in response to a variety of stress stimuli (22).

The Apaf-1 gene encodes a cytoplasmic protein, which presents one of the most important factors in the apoptosis regulatory network. The Apaf-1 protein binds and cleaves caspase-9 preproprotein, releasing the mature, activated caspase-9, which stimulates a subsequent caspase cascade that causes the cancer cell to undergo apoptosis (23). Caspase-8 initiates disassembly in response to signals from extracellular apoptosis-inducing ligands (24). Caspases present a proteolytic network within the cell in which upstream initiator caspases are activated early in the apoptotic process (caspase-9), leading to the activation of downstream caspases (caspase-3). Caspase-3 amplifies caspase-9 and caspase-9 initiation signals to induce nuclear disassembly (25).

Fas is a death domain-containing member of the tumor necrosis factor receptor superfamily, which is associated with apoptosis of cancer cells. The FasL-Fas system has been investigated with respect to its death-inducing function. The Fas receptor exerts an apoptotic signal by binding to FasL, which is expressed on the surface of other cells (26). FasL signals via trimerization of FasR, which spans the membrane of the target cell. This trimerization usually leads to apoptosis (27).

In the death receptor pathway, a signaling cascade leads to the activation (using an adaptor, Fas-associated protein with death domain) of a caspase cascade involving caspase-8 and -3. BH3-only proteins are firstly upregulated or activated by cytotoxic injury during the mitochondrial pathway, and subsequently activate and oligomerize Bax, which leads the oligomerized Bcl-2 family members, Bax/Bak, to induce the release of cytochrome *c* from the mitochondria into the cytosol. Consequently, cytochrome *c* and Apaf-1 form a complex, the apoptosome, which activates caspase-9 and subsequently caspase-3 (28). In agreement with the results of the present study, the induction of apoptosis in drug or functional material-treated cancer cells has been reported to increase Bax, p53, p21, Apaf-1, caspase-3,-8,-9 and Fas gene expression, and decrease Bcl-2, Bcl-xL and FasL gene expression (9,29–32).

In the present study, PLCSB induced a high level of apoptotic activity in HCT-116 colon cancer cells, *in vitro*. PLCSB, particularly at high concentrations, induced apoptosis, which was demonstrated by DAPI staining, flow cytometry analysis, and changes in the mRNA and protein expression of apoptosis-related genes. PLCSB may be used as health product or medicine for cancer prevention and treatment in the future.
